# Fatal *Chromobacterium violaceum *septicaemia in northern Laos, a modified oxidase test and post-mortem forensic family G6PD analysis

**DOI:** 10.1186/1476-0711-8-24

**Published:** 2009-07-29

**Authors:** Günther Slesak, Phouvieng Douangdala, Saythong Inthalad, Joy Silisouk, Manivanh Vongsouvath, Amphonesavanh Sengduangphachanh, Catrin E Moore, Mayfong Mayxay, Hiroyuki Matsuoka, Paul N Newton

**Affiliations:** 1 SFE Medical Project, P.O. Box 56, Luang Namtha, Lao People's Democratic Republic; 2 Luang Namtha Provincial Hospital, Luang Namtha, Lao People's Democratic Republic; 3 Wellcome Trust-Mahosot Hospital-Oxford Tropical Medicine Research Collaboration, Microbiology Laboratory, Mahosot Hospital, Vientiane, Lao People's Democratic Republic; 4 Centre for Clinical Vaccinology and Tropical Medicine, Churchill Hospital, University of Oxford, Oxford, England, UK; 5 Faculty of Post-Graduate Studies and Research, University of Health Sciences, Vientiane, Lao PDR; 6 Division of Medical Zoology, Department of Infection and Immunity, Jichi Medical University, Japan

## Abstract

**Background:**

*Chromobacterium violaceum *is a Gram negative facultative anaerobic bacillus, found in soil and stagnant water, that usually has a violet pigmented appearance on agar culture. It is rarely described as a human pathogen, mostly from tropical and subtropical areas.

**Case presentation:**

A 53 year-old farmer died with *Chromobacterium violaceum *septicemia in Laos. A modified oxidase method was used to demonstrate that this violacious organism was oxidase positive. Forensic analysis of the glucose-6-phosphate dehydrogenase genotypes of his family suggest that the deceased patient did not have this possible predisposing condition.

**Conclusion:**

*C. violaceum *infection should be included in the differential diagnosis in patients presenting with community-acquired septicaemia in tropical and subtropical areas. The apparently neglected but simple modified oxidase test may be useful in the oxidase assessment of other violet-pigmented organisms or of those growing on violet coloured agar.

## Background

*Chromobacterium violaceum *is a Gram negative facultative anaerobic bacillus, found in soil and stagnant water, that usually has a violet pigmented appearance on agar culture. Since its discovery as a human pathogen in 1927 in Malaysia [1] only ~150 human cases have been reported worldwide, mostly from tropical and subtropical areas. *C. violaceum *septicaemia has been described in Thailand, with two patients from a province adjacent to Laos [2] and Vietnam [3,4] but, to our knowledge, this is the first patient described in Laos. The disease typically starts with a localized skin infection or localized lymphadenitis after contact with stagnant water or soil and progresses to fulminating septicaemia with necrotizing metastatic lesions and multiple abscesses in the liver, lung, spleen, skin, lymph nodes, and brain, resulting in fatal multiorgan failure [1-10]. The mortality from disseminated *C. violaceum *infection has been reported to be 60–80% [1-10]. However, the optimal treatment has not been established – combination therapy with co-trimoxazole, chloramphenicol, carbapenems, or fluoroquinolones has been suggested [2,7,9]. Resistance to penicillins and cephalosporins has been commonly reported [3,9,11] and makes therapy while microbiology results are awaited difficult as both antibiotics are commonly used for empirical therapy of septicemia.

## Case presentation

A 53-year-old Tai Dam [12] Lao farmer was admitted in August 2008 at the Provincial Hospital of Luang Namtha, northern Lao PDR (Laos), with 2 days of fever, chills, severe headaches, nausea and vomiting, and a cough productive of white sputum. He had been healthy apart from serious leg injuries from a relict bomb explosion 6 years previously. On admission he was alert and orientated, normotensive, without neck stiffness, abdominal tenderness, lymphadenopathy, hepatosplenomegaly, pallor or jaundice but was febrile (39.7°C) with crepitations audible at the left lung base. He had no rash but old scars on his thighs from the bomb injury and minor scars on his lower legs and flanks from insect bites. He was not known to contain shrapnel. With suspected community-acquired pneumonia he was started on oral amoxicillin (1 g twice daily) and paracetamol. With an increase of his fever to 40.2°C, metamizole (1 g twice daily IV) was added. On day 2 his full blood count showed normochromic anaemia (Hct 18%, Hb 6 g/dL, MCHC 33 g/dL, leucocytes 7.5 10^9^/L, neutrophils 70%, lymphocytes 30%). Malaria smear and HRP-2 rapid test for *Plasmodium falciparum *malaria (Paracheck™, Orchid Industries, Goa, India) were negative. An abdominal ultrasound demonstrated a diffuse enlarged liver, without focal lesions, and mild splenomegaly. A chest radiograph showed a slightly enlarged heart but no infiltrations. The patient developed upper abdominal and lower back pain and increasing dyspnoea and was pale and jaundiced with a respiratory rate of 42/min, temperature 39°C, blood pressure 90/60 mmHg, heart rate irregular 120/min, bibasal inspiratory crepitations, tender hepatomegaly (1 cm below costal margin in mid-clavicular line) with axillary and inguinal lymphadenopathy. As community-acquired septicaemia or leptospirosis were suspected, therapy was changed to ceftriaxone (1 g twice daily) and he was given a blood transfusion. However, he deteriorated with worsening abdominal pain and distension, dyspnoea, central cyanosis with cold extremities, coma (GCS 7/15) and a disseminated maculopapular rash with petechiae on arms and trunk and prolonged bleeding at intravenous injection sites. On day 3 he became apnoeic and could not be resuscitated.

**Figure 1:**
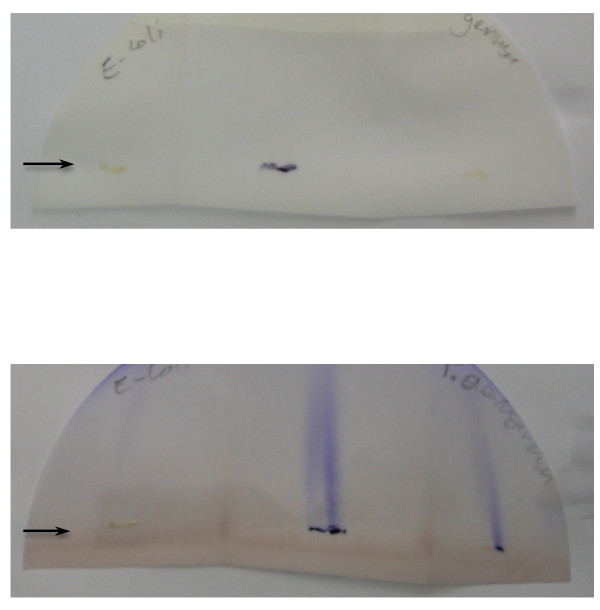
**Oxidase test adapted from Dhar & Johnson **[15]. Smears (arrows) of organisms were applied to two Whatman 3 M filterpapers folded, vertically, into three. The filter papers were placed vertically in Petri dishes so that the solution within soaked vertically across the smears of organisms. Top panel: filterpaper stood in 10 ml distilled water in a Petri dish, bottom panel: filterpaper stood in 10 ml 1% aqueous solution of N, N, N', N'-tetramethyl-1, 4-phenylene-diamine dihydrochloride (Fluka 87890, Czech Republic) in a Petri dish. The organisms smeared were left *E. coli *ATCC 25922 (negative control), centre *C. violaceum *isolate from patient and right *P. aeruginosa *ATCC 27853 (positive control). Water (top panel) failed to produce any streaks whilst N,N,N',N'-tetramethyl-1, 4-phenylene-diamine dihydrochloride (bottom panel) led to pronounced vertical flame-like streaks across smears of *C. violaceum *and *P. aeruginosa *within 5 minutes, indicating oxidase test positivity. The flame-like streaks had faded by the next day. The profound violet color of the *C. violaceum *smear is clearly visible.

Blood cultures from admission and day 2, sent by plane to Vientiane [13,14], grew *Chromobacterium violaceum *(Gram negative rods, catalase positive, Voges-Proskauer test negative, violacious colour on agar, API 20 NE (bioMerieux, France) 99.3% agreement). As the *C. violaceum *were pigmented violet conventional oxidase tests could not be interpreted and the ingenious but apparently neglected method of Dhar & Johnson [15] was used to demonstrate that the organism was oxidase positive (Fig. 1). The organism was sensitive to gentamicin, chloramphenicol, ofloxacin, ciprofloxacin, co-trimoxazole, imipenem, and resistant to ampicillin, ceftriaxone, and ceftazidime by disc diffusion testing (using NCCLS criteria for *Vibrio *or Enterobacteracea). Etest (AB Biodisk, Solna, Sweden) demonstrated MICs of 0.023, 4 and >32 ug/L against ofloxacin, chloramphenicol and ceftriaxone, respectively. After his death his family was re-interviewed regarding possible risk factors. He had been pale with mild jaundice since childhood and when aged ~12 years was said to have had a big spleen. One week before the onset of fever he had gone to rice fields and fishing and slept at a market where he noted an axillary leech. The bite wound did not become overtly infected.

Glucose-6-phosphate dehydrogenase (G6PD) deficiency has been suggested as a risk factor for *C. violaceum *infection [7,16] but the aetiological diagnosis was only made after the patient's death and no blood samples were available for G6PD testing. Therefore, with their informed consent, his wife and 4 daughters were tested for G6PD deficiency, using finger prick blood samples on filter paper, by quantitative spectrophotometric analysis (WST-8 method [17]) and sequencing of the G6PD gene in the X-chromosome [18]. The patient's wife and 3 of the 4 tested daughters had a substitution of G to A at the 1388 position in G6PD gene, signifying carriage of G6PD Kaiping. Since one of the daughters had wild type X-chromosomes with normal G6PD activity the patient evidently did not have G6PD deficiency.

Our patient did not have a primary skin infection. However, he had a leech bite a week before onset of fever which may or not have been significant – as far as we are aware this organism has not been described from the leech gastrointestinal tract. The patient's striking anaemia, severe abdominal and lower back pain, tachycardia, and jaundice in combination with a history of pallor and splenomegaly in childhood could be explained by an acute intravascular haemolysis due to underlying G6PD deficiency, possibly precipitated by metamizole (dipyrone), which is contraindicated in G6PD deficiency [19]. Laos has a high prevalence of G6PD deficiency (15–26% of males hemizygous [20,21]). In view of G6PD being a possible risk factor for both his infection and haemolysis we performed forensic analysis of the surviving members of his family. However, this demonstrates, assuming correct blood relationships, that he could not have had G6PD deficiency. G6PD Kaiping is a common mutation in neighboring China [22].

Melioidosis (*Burkholderia pseudomallei*) is a common and frequently fatal condition in Northeast Thailand and the Mekong valley of Laos [14] but has, so far, not been described from the highlands of northern Laos and it may not occur in the soil there. *C. violaceum *and melioidosis resemble each other with reservoirs in soil and water, clinical presentation, resistance to antibiotics commonly used to empirically treat septicaemia, high mortality and the need for prolonged oral antibiotic eradication therapy. However, as *C. violaceum *is frequently resistant to cephalosporins, such as ceftazidime [5,9], commonly used to treat melioidosis, awareness of *C. violaceum *as an alternative diagnosis is important [2]. The two organisms can be confused in the laboratory and both are oxidase positive [23]. The ingenious method of Dhar & Johnson [15] was used to determine the oxidase status of the organism – this apparently neglected but simple technique may be useful in the oxidase assessment of other violet-pigmented organisms or of those growing on violet colored agar. A similar method was described [24] after that of Dhar & Johnson [15]. Conventional oxidase testing of very young or anaerobic *C. violaceum *cultures, which are usually non-pigmented, have also been used but the evidence available suggests that they may not be accurate [6,24].

## Conclusion

*C. violaceum *infection should be included in the differential diagnosis of patients presenting with community-acquired septicaemia in tropical and subtropical areas, especially with a history of contact with soil and stagnant water, and is an important alternative diagnosis for patients presenting with a melioidosis-like syndrome.

## Competing interests

The authors declare that they have no competing interests.

## Authors' contributions

GS, PD, SI were the attending physicians who looked after the patient. JS, MV, PNN, CEM and MM preformed the microbiological examinations. PD, GS did the home visits of the patient's family and HM the G6PD assays and sequencing. GS and PNN wrote the first draft and all authors revised it. All authors have read an approved the final version.

## Consent

Written informed consent was obtained from the patient's family for publication of this case report and the forensic investigation.

## Financial support

We thank the Wellcome Trust of Great Britain for financial support of the microbiology testing and SFE for the logistical support of the specimen transport.
